# Supplementary material for: Prevalence and Risk Factors Associated with Multidrug Resistance and Extended-Spectrum β-lactamase Producing E. coli Isolated from Healthy and Diseased Cats
Source: Antibiotics (Basel). 2023 Jan 20;12(2):229. doi: 10.3390/antibiotics12020229 (PMC9951988; doi:10.3390/antibiotics12020229)
Supplement: Supplementary file 1 [file antibiotics-12-00229-s001.zip › antibiotics-2135141-supplementary.pdf]

Table S1: Primers, product size and annealing temperatures used in the present study to identify virulence and antimicrobial resistance genes.

Gene	Primer sequences	Product size (bp)	Annealing (°C)	References
$bla_{\mathrm{CTX\text{-}M\text{-}1}}$	fw: GAC GAT GTC ACT GGC TGA GC<br>rev: AGC CG C CGA CGC TAA TAC A	499	55	[65]
$bla_{\mathrm{CTX\text{-}M\text{-}2}}$	fw: GCG ACC TGG TTA ACT ACA ATC C<br>rev: CGG TAG TAT TGC CCT TAA GCC	351	55	
$bla_{\mathrm{CTX\text{-}M\text{-}3}}$	fw: CGC TTT GCC ATG TGC AGC ACC<br>rev: GCT CAG TAC GAT CGA GCC	307	55	
$bla_{\mathrm{CTX\text{-}M\text{-}4}}$	fw: GCT GGA GAA AAG CAG CGG AG<br>rev: GTA AGC TGA CGC AAC GTC TG	474	62	
bla_{SHV}	fw: CACTCAAGGATGTATTGTG<br>rev: TTAGCGTTGCCAGTGCTCG	885	50	[63]
bla_{TEM}	fw: TCGGGGAAATGTGCGCG<br>rev: TGCTTAATCAGTGAGGCACC	971	50	
stx1	fw: AAATCGCCATTCGTTGACTACTTCT<br>rev: TGCCATTCTGGCAACTCGCGATGCA	370	60	[62]
stx2	fw: CAGTCGTCACTCACTGGTTTCATCA<br>rev: GGATATTCTCCCCACTCTGACACC	283	60	
eaeA	fw: CCCGAATTCGGCACAAGCATAAGC<br>rev: CCCGGATCCGTCTCGCCAGTATTCG	863	52	[64]
hlyA	fw: GGTGCAGCAGAAAAAGTTGTAG<br>rev: TCTCGCCTGATAGTGTTTGGTA	1551	57	

Table S2. Description of variables collected from both healthy and diseased cats.

Factors	Categories	Total	Healthy cats (n = 209)		Diseased cats (n = 191)	
			Frequency	%	Frequency	%
**Cat sex**						
	Female	227	131	62.7	96	50.3
	Male	173	78	37.3	95	49.7
**Cat breed**						
	Himalayan	59	15	7.2	44	23.0
	Persian	162	106	50.7	56	29.3
	Birman	53	21	10.1	32	16.8
	Siamese	112	64	30.6	48	25.1
	Egyptian Mau	10	3	1.4	7	3.7
	Arabian Mau	4	--	--	4	2.1
**Family use antimicrobials**						
	No	308	177	84.7	131	68.6
	Yes	92	32	15.3	60	31.4
**Family member with acne**						
	No	282	150	71.8	132	69.1
	Yes	118	59	28.2	59	30.9
**Hospitalization**						
	No	334	188	89.9	146	76.4
	Yes	66	21	10.1	45	23.6
**Previous antimicrobial use for cat**						
	No	238	144	68.9	94	49.2
	Yes	162	65	31.1	97	50.8
**Current antimicrobials use for cat**						
	No	384	208	99.5	176	92.2
	Yes	16	1	0.5	15	7.8
**Child at home**						
	No	205	99	47.4	106	55.5
	Yes	195	110	52.6	85	44.5
**Cat living**						
	Indoors	133	78	37.3	55	28.8
	Indoors-outdoors	267	131	62.7	136	71.2
**Reason being at clinic**						
	Vaccination and/or grooming	209	207	99.0	2	1.1
	Treatment	191	2	1.0	189	98.9
**Cat care**						
	Adult male	116	53	25.4	63	33.0
	Adult female	192	93	44.5	99	51.8
	Child	18	12	5.7	6	3.1
	All family	74	51	24.4	23	12.1
**diet type**						
	Dry	263	137	65.6	126	66.0
	Semi-moist	2	2	1.0	--	--
	Wet	53	23	11.0	30	15.7
	Raw uncooked	35	30	14.3	5	2.6
	Home available	47	17	8.1	30	15.7
**Total**		**400**	**209**	**52.3**	**191**	**47.7**
